# Design of Advanced Photocatalysis System by Adatom Decoration in 2D Nanosheets of Group-IV and III–V Binary Compounds

**DOI:** 10.1038/srep23104

**Published:** 2016-03-17

**Authors:** Hao Jin, Ying Dai, Bai-Biao Huang

**Affiliations:** 1 College of Physics and Energy, Shenzhen University, Shenzhen 518060, People’s Republic of China; 2 School of Physics, State Key Laboratory of Crystal Materials, Jinan, 250100, People’s Republic of China

## Abstract

Searching for novel photocatalysts is one of the most important topic in photocatalytic fields. In the present work, we propose a feasible approach to improve the photocatalytic activities of 2D bilayers through surface decoration, i.e. hydrogenation, halogenation, and hydroxylation. Our investigations demonstrate that after surface modification, the optical adsorption expands into the visible region, while a built-in electric field is induced due to the interlayer coupling, which can promote the charge separation for photogenerated electron-hole pairs. Our results show that the indirect-direct band gap transition of SiC, SnC, BN and GaN can be realised through adatom decoration. Furthermore, the surface-modified 2D bilayers have suitable VBM and CBM alignments with the oxidation and reduction potentials for water splitting, suggesting powerful potentials in energy and environmental applications.

Stimulated by the research on graphene, an intense research interest has been focused on two-dimensional (2D) materials, which has opens up a new way to searching for efficient composite photocatalysts^1^,^2^,^3^,^4^. Over the past few years, a large variety of graphene-analogous materials such as BN, C_3_N_4_, and dichalcogenides (e.g. MoS_2_ and WSe_2_) have been explored and studied, and promising properties are reported^5^,^6^,^7^,^8^,^9^. For example, Wang *et al.* shown that the hybrid graphene-like C_3_N_4_ and ZnO is a good candidate of photocatalysis and exhibits enhanced stability against photocorrosion^7^. Similarity, Zhou and co-workers demonstrated that a TiO_2_ photocatalyst hybridized with MoS_2_ monolayer exhibits high photocatalytic efficiency for water splitting, giving the hydrogen production rate up to 1.6 mmolh^−1^g^−1^ 8. Very recently, Zhang and Xu reviewed the recent achievements of graphene-semiconductor composite photocatalysts, and highlighted that the graphene-semiconductor composite photocatalysts shown high efficiency in solar energy conversion^10^,^11^. Nevertheless, most of the 2D materials with honeycomb structures, such as carbides XC (X = Si, Ge, Sn) of group IV and nitrides YN (Y = B, Ga) of group III–V, are not suitable for photocatalytic process due to either large band gaps or inefficient photogenerated charge carriers separations^12^. Consequently, designing and modifying graphene-like 2D materials that exhibits desirable physical and chemical properties is an urgent task and a great challenge.

In 2D nanosheets, atoms which are exposed on the surface have high chemical activities. And consequently, surface chemical modification could trigger lattice variation and charge transfer, which has a great influence on the whole electronic state of the 2D materials. For example, hydrogenation could modulate graphene from the semi-metal to an electronic insulator^13^,^14^. The introduced magnetism is also observed in surface-decorated graphene and other 2D nanosheets^15^,^16^,^17^,^18^. Recently, Lin *et al.* developed the unprecedentedly high electrical conductivity (6.76 × 10^4^ S/m at room temperature) in metallic 2D nanomaterials via hydrogenation, which is the best value reported among the graphene and graphene analogues 2D materials^19^. In addition, the strategies to fabricate semi-hydrogenated 2D materials are also explored^20^,^21^. Therefore, surface modification strategies have been considered as an important and feasible method to tune the intrinsic physical properties of 2D materials^22^,^23^,^24^,^25^.

Despite extensive studies on conductivity and magnetism, less attention is allocated on the photocatalytic activity of surface modified 2D materials^26^,^27^. In addition, the surface effects on light absorption efficiency and carrier separation are still not clear yet. Therefore, it is of great interest to study the influence of surface modification on the photocatalytic efficiency of these 2D nanosheets. In this study, based on the hybrid density functional calculations with the inclusion of the nonlocal van der Waals (vdW) correction, we systematically investigated the photocatalytic activities of carbides XC (X = Si, Ge, Sn) of group IV and nitrides YN (Y = B, Ga) of group III–V 2D materials after surface decoration, that is, hydrogenation, halogenation, and hydroxylation. Our results demonstrate that surface modification can not only enhance the optical response to the visible light region, but also promote the charge separation for photogenerated electron-hole pairs, enabling the development of more efficient visible-light driven photocatalysts.

## Results

As a starting point, we first present a detailed analysis of GeC, which has a planar hexagonal structure due to the *sp*^2^ hybridization^12^. The optimized cell parameter is found to be 3.26 Å with the Ge-C bond distances of 1.883 Å. From the GGA calculations, bare GeC sheet is found to have a direct band gap of 1.88 eV. To correct the underestimation of the standard DFT calculations, HSE06 hybrid functional is used for comparison. The band gap calculated through the HSE06 functional is found to be 3.21 eV, which is consistent with previous studies^28^.

The band gap of GeC sheet is so large for solar energy conversion. To improve light absorption and photocatalytic performance, we design a promising system using surface-functionalized GeC bilayer. In this structure, the GeC bilayer possesses a low-energy AB stacking arrangement. The surface decoration can be fulfilled by absorbing A (A = H, OH, F, Cl, and Br) atoms on carbon site or germanium site. To determine the most stable structure, we studied the formation energies of surface decorated GeC bilayer by calculating:


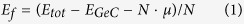


where *E*_*tot*_ is the energy of the surface-decorated GeC bilayer, *E*_*GeC*_ is the energy of pure GeC bilayer, *μ* represents the chemical potential of adatom, and N is the number of adsorbed atoms.

**Figure 1:**
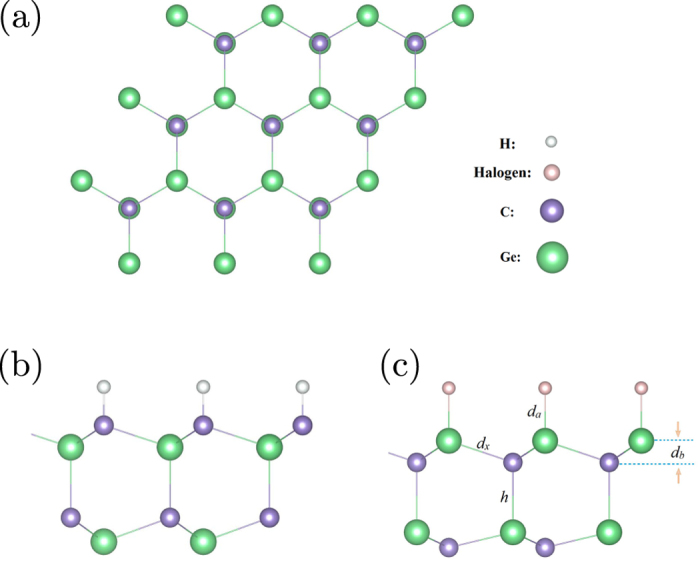
(**a**) Top view of GeC bilayer; Side view of (**b**) hydrogenated and (**c**) halogenated GeC bilayers.

Our results show that hydrogen prefers to adsorb on carbon sublattice, while OH and halogen prefer to adsorb on Ge sublattice. The optimized atomic configuration from top and side views are shown in Fig. 1. It is noted that surface decoration changes the graphitic GeC sheet from a planar structure to a buckled one. The buckling parameter *d*_*b*_ varies from 0.59 Å to 0.64 Å depending on the adatom. The equilibrium interlayer distance *h* is defined as the distance between C atoms and Ge atoms in different layers. The calculated bilayer distance *h* for surface-modified GeC bilayer is considerable smaller than the value of bare GeC bilayer, i.e. 3.26 Å, suggesting higher adhesion energy. The corresponding structural parameters are listed in Table 1.

The adatoms possess strong electron affinity, enabling them gain electrons from GeC sheets. When hydrogen (OH, halogen) adsorbed on GeC bilayers, each C-H (Ge-OH, Ge-halogen) bond generates one hole and therefore surface-decorated GeC can exhibit magnetism. To study the ground state of the surface-decorated GeC, three magnetic configurations are examined: (1) ferromagnetic (FM) coupling; (2) antiferromagnetic (AFM) coupling; and (3) nonmagnetic (NM) state. The calculated energy differences (Δ*E*_*m*_) between AFM and FM states are listed in Table 1. It is found that in all cases, the ground state is FM state. The NM states are much less stable than the FM states with an energy difference up to 0.6 eV.

**Figure 2:**
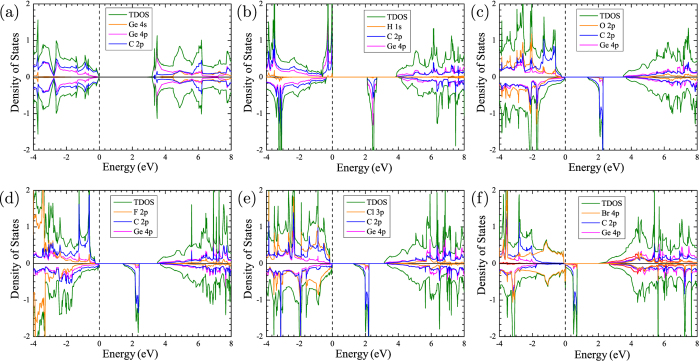
Density of states of (**a**) GeC, (**b**) GeC/GeC-H, (**c**) OH-GeC/GeC, (**d**) F-GeC/GeC, (**e**) Cl-GeC/GeC and (**f**) Br-GeC/GeC. The Fermi level is indicated by the dashed line.

The calculated density of states (DOS) of surface-decorated GeC bilayers are shown in Fig. 2. It is clearly shown that the valence band maximum (VBM) of GeC sheet is mainly contributed by C 2*p*-orbital, whereas the conduction band minimum (CBM) is mainly composed of Ge 4*p* states. When GeC sheets are functionalized by hydrogen, the unpaired *p*-electrons of Ge atoms produce new energy states in the middle of the band gap, resulting in the magnetism of the system. In the same way, for OH and halogen modified GeC bilayers, the energy states of C 2*p*-orbitals locates above the VBM in the range of 0:1.43 eV. More interestingly, we found that the unpaired C 2*p*-states shift towards the VBM depending on the electronic affinity of the adatom. Especially, for Br-GeC/GeC bilayer, the VBM is mixed with C 2*p*-orbitals. As a result, the band gap of surface-modified GeC bilayers is significantly narrowed, which is greatly favorable for the adsorption of the visible light.

**Figure 3:**
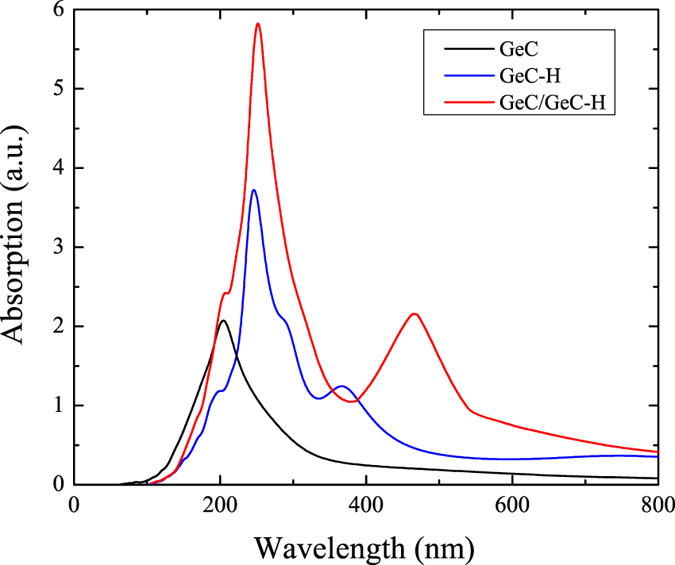
The calculated optical absorption for GeC monolayer, GeC-H monolayer and GeC/GeC-H bilayer as a function of wavelength.

In order to investigate the visible-light adsorption ability of surface modified GeC bilayer, we calculated the optical absorption spectrum of GeC/GeC-H sheet, which is obtained by Equation 5. As shown in Fig. 3, we found that GeC/GeC-H bilayer shows an enhanced absorption, with the integrated intensity up to four times larger than the GeC monolayer, and two times larger than the GeC-H monolayer. In addition, the absorption spectrum of such hybrid complex has been expanded into the yellow-orange region of the visible spectrum comparing with the pure GeC monolayer, indicating that the hydrogenated GeC bilayer could harvest a broader range of visible light efficiently.

**Figure 4:**
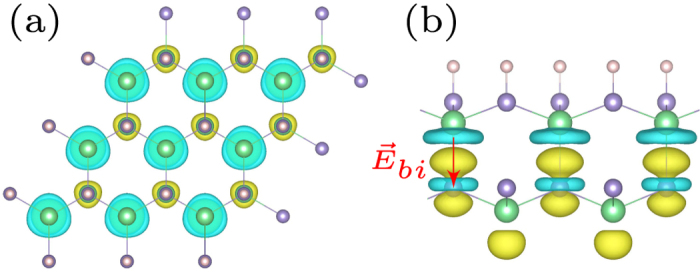
(**a**) Top and (**b**) side view of the charge density difference at the GeC/GeC-H bilayer. The light blue and yellow isosurfaces indicate charge depletion and accumulation with isovalue of 0.005 e/Å^3^.

From Fig. 3, one can see that in comparison with the single layer, GeC/GeC-H bilayer has much better visible light adsorption efficiency, which can be ascribed to the interlayer coupling. In fact, the surface modified GeC bilayer can be considered as the GeC/GeC-H heterointerface. To get a further insight, the charge density difference is plotted in Fig. 4, which is obtained by subtracting the charge density of the GeC/GeC-H bilayer from that of the independent GeC-H and GeC monolayers. It can be seen clearly that there is a space charge region within the GeC/GeC-H heterointerface. This can be understood based on the fact that after surface hydrogenation, the unpaired Ge 2*p*-electrons in the top layer are strongly attracted by the carbon atoms in the bottom layer. The interaction between Ge and C atoms exerts a driving force, which leads to the electrons transfer from the areas of Ge atoms to the carbon region, while holes move in the opposite way.

Using Bader charge analysis, we obtained the values for the charge transfer from Ge to the carbon atoms. We found that charge transfer across the heterointerface occurs on GeC-GeCH bilayer (see Fig. S1 of supplementary information). A net charge gain at the GeC side has been identified with C-2*p* orbitals being the electron accommodator. While each Ge atom loses 0.26 e in the GeC-H sheet. As shown in Fig. 4(b), when the charge redistribution in the GeC/GeC-H heterointerface reaches equilibrium, a built-in electric field (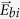) is induced. This hetero-bilayer is a typical type-II hetero-junction: the position of conduction and valence band edges of hydrogen modified GeC monolayer is respectively lower than that of bare GeC monolayer, which leads to band bending between top and bottom monolayers.

**Figure 5:**
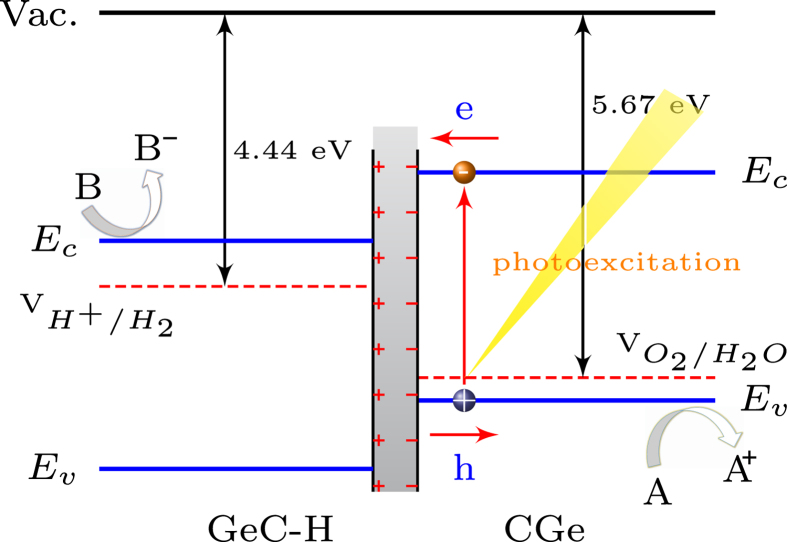
The Band alignment at GeC/GeC-H interface relative to the vacuum energy.

As illustrated in Fig. 5, the built-in-field can drive the photo-generated electrons and holes move in opposite direction. The characteristics of built-in-field can help to introduce spatial separation of the electrons and holes on different layers. Thus, the formation of type-II GeC/GeC-H heterointerface is an effective approach to enhance charge-hole separation and reduce their recombination, which is useful for photronic devices or photocatalytic technology.

In addition to an appropriate band gap, a good photocatalyst material generally needs suitable VBM and CBM energy levels. In this study, we determine the band edges via the so-called potential-line-up method involving the macroscopic averaging of the electrostatic potential^29^,^30^. The macroscopic average potential 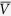, which was proposed by Baldereschi *et al.*^29^ is defined as:


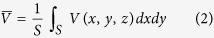


where 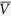 is the plane-averaged electrostatic potential and *S* represents the area of the interface.

As shown in Fig. 5, accurate energy locations of VBM and CBM with respect to the redox potentials of water splitting are determined. On the GeC surface, the oxidation potential (5.67 eV) of 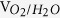 is higher than VBM, suggesting photogenerated holes can be readily transferred from valence band to H_2_O, oxidizing it to O_2_ through the reaction:


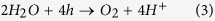


Meanwhile the CBM energy of bottom GeC-H layer lies above the reduction potential (4.44 eV) of H^+^/H_2_ to further produce H_2_:


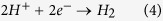


**Figure 6:**
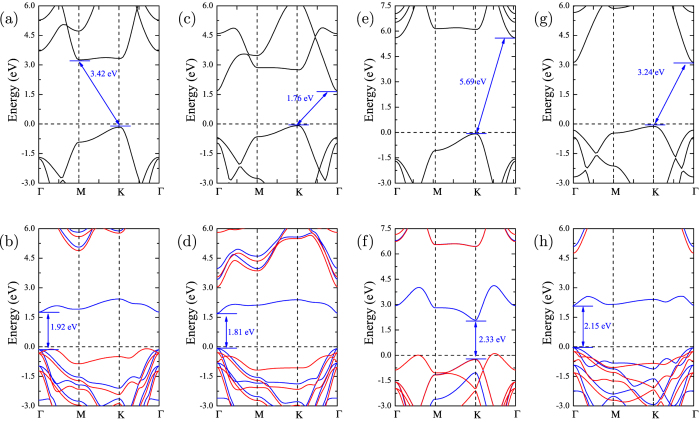
The calculated band structures for (**a**) SiC, (**b**) H-SiC, (**c**) SnC, (**d**) H-SnC, (**e**) BN, (**f**) H-BN, (**g**) GaN and (**h**) H-GaN nanosheets with HSE06 functional. The spin-up and spin-down states are demonstrated with red and blue lines, respectively. The Fermi level is indicated by the dashed line.

Therefore, hydrogenated GeC bilayer acts as a promising visible-light-driven photocatalyst for water splitting. In addition to GeC, we also examine the related properties of other 2D nanosheets of group-IV and III–V binary compounds, SiC, SnC, BN, and GaN. As discussed above, the photocatalytic activities of these 2D materials are hindered by either large band gaps or inefficient photogenerated electron-hole separations (see Fig. 6). For example, the band gap of pure BN sheet is up to 5.69 eV, which is too large for light adsorption. However, when hydrogen adsorbed on the surface, it shows that these 2D materials become potential photocatalysts with their band gaps ranging from the visible light region to the near-infrared light region.

**Figure 7:**
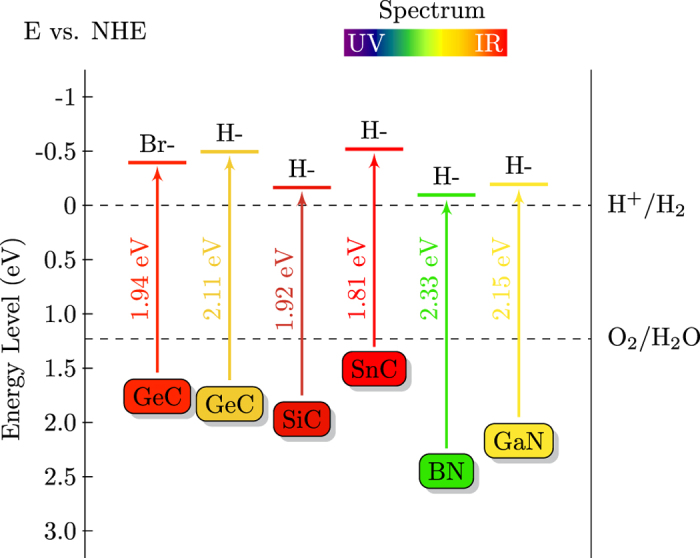
Calculated edge positions of VBM and CBM for surface modified 2D materials relative to the vacuum energy.

Moreover, from Fig. 6 we can see that SiC, SnC, BN and GaN nanosheets are indirect band gap materials. The VBM locates at the K point, while the CBM locates at either M point (SiC) or Γ point (SnC, BN, and GaN). By surface decoration, the transition between indirect and direct band gaps nanosheets are observed. The direct-band-gap hydrogenated 2D bilayers are expected to have high solar energy conversion efficiency compared to those indirect-band-gap monolayers. In Fig. 7, the band gap energy and the corresponding CBM and VBM edge positions versus NHE are plotted. It can be clear seen that for all cases, the reduction level is below the CBM, while the oxidation level is above the VBM. This reveals the oxidation and reduction processes are energetically favored with a relatively strong driving force, suggesting that the hydrogenated 2D bilayers are good candidates as photocatalysts for water splitting.

## Discussion

Owing to the dimensionally reduced structure where atoms are exposed on the surface with high chemical activity, regulating band gap and carrier separation of 2D materials were achieved by surface chemical modifications. The structural and electronic properties of carbides XC (X = Si, Ge, Sn) of group IV and nitrides YN (Y = B, Ga) of group III–V are carefully investigated through hybrid functional DFT calculations with the inclusion of the nonlocal vdW correction. Our results show that after surface modification, the band gaps of such graphene-like 2D materials are significantly narrowed, which corresponds to the visible spectrum. Note that the states introduced by the adatoms are no longer the gap states, but form the new states, i.e. the conduction band of the system. In addition, since atoms are exposed on the surface with high chemical activities, the redox reactions occur with these surface atoms. So unlike the bulk cases, the electrons cannot be trapped anymore, but rather to participate in the chemical reactions directly. Moreover, the couplings within the surface-modified bilayers lead to the charge redistribution, which gives rise to the band bending. As a result, a built-in electric field creates, and a type-II hetero-junction forms within the interlayer. Further analysis indicates that such heterointerface could be beneficial to facilitate the photogenerated charge separation, and enhance the optical response under visible-light region. In addition, a transition between indirect and direct band gaps of SiC, SnC, BN and GaN are observed, which is greatly favorable for the adsorption of the visible light.

In this viewpoint, 2D nanomaterials provide an ideal platform for promoting photocatalytic efficiency via surface modification. Note that the surface functionalization is not limited to hydrogenation, halogenation, and hydroxylation as discussed in this study, but can be applied to many other methods. For example, small functional group, i.e. NH_3_, intrinsic defects^25^,^31^,^32^,^33^, and even isolated metal atom, which is known as single atom catalyst, and has been found to show excellent stability and high activity in metal surfaces^34^,^35^. Thus, from our results, it is expected that surface modification of 2D nanosheets will lead to the discovery of new promising visible-light driven photocatalysts.

## Methods

Spin-polarized DFT calculations were performed using the Vienna *ab initio* simulation package (VASP)^36^,^37^. Projector augmented wave (PAW)^38^ were used from the standard VASP library with the Perdew-Burke-Ernzerhof (PBE) generalized gradient approximation (GGA) exchange correlation functional^39^. Ground state structures were found by allowing all systems to fully relax with respect to both atomic positions and supercell shape and size using a conjugate-gradient method with energy convergence of 10^−6^ eV and force convergence of 10^−3^ eV/Å. The cutoff energy for the basis set was chosen to be 500 eV, and the Brillouin zone was sampled with Monkhorst-Pack grid of 18 × 18 × 1^40^. The vacuum space is set to be 20 Å, which gives well-converged results.

The state-of-the-art hybrid functional (HSE06) is used for all calculations^41^,^42^, in which the hybrid functional is mixed with 25% exact Hartree-Fock (HF) exchange. A damped vdW correction based on Grimme’s scheme is also incorporated to better describe the nonbonding interaction^43^.

The absorption coefficient (*α*(*E*)) is evaluated according to the following expression:^5^


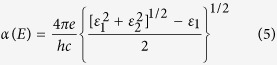


where *ε*_1_ and *ε*_2_ represent the real and imaginary parts of the dielectric function, respectively. The imaginary part is calculated by a summation over empty states using the equation:^44^


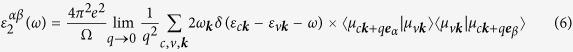


where the indices *c* and *v* refer to conduction and valence band states, respectively. *μ*_*ck*_ represents the cell periodic part of the wavefunctions at the *k* point. The real part of the dielectric tensor *ε*_1_(*ω*) is obtained by a Kramers-Kronig transformation.

## Additional Information

**How to cite this article**: Jin, H. *et al.* Design of Advanced Photocatalysis System by Adatom Decoration in 2D Nanosheets of Group-IV and III–V Binary Compounds. *Sci. Rep.* **6**, 23104; doi: 10.1038/srep23104 (2016).

## Supplementary Material

Supplementary Information

## Figures and Tables

**Table 1:** Calculated lattice constant, *a*; distance between Ge and adatom, *d*_*a*_; nearest neighbor distance, *d*_*x*_; buckling parameter, *d*_*b*_; bilayer distance, *h*; Energy difference between AFM and FM states, Δ*Em*; and band gaps, *E*_*g*_.

	*a* (Å)	*d*_*a*_ (Å)	*d*_*x*_ (Å)	*d*_*b*_ (Å)	*h* (Å)	Δ*E*_*m*_ (meV)	*E*_*g*_ (eV)
GeC	3.26	–	1.883	0.000	–	–	3.21
GeC/GeC-H	3.29	1.567	2.006	0.638	2.020	50	2.11
OH-GeC/GeC	3.33	1.823	2.008	0.609	2.076	90	1.36
F-GeC/GeC	3.34	1.763	2.018	0.593	2.079	90	1.43
Cl-GeC/GeC	3.38	2.160	2.045	0.611	2.079	91	1.26
Br-GeC/GeC	3.32	2.357	2.008	0.599	2.111	98	1.94

The structural parameters are described in Fig. 1.
