# Supplementary material for: Design of Advanced Photocatalysis System by Adatom Decoration in 2D Nanosheets of Group-IV and III–V Binary Compounds
Source: Sci Rep. 2016 Mar 17;6:23104. doi: 10.1038/srep23104 (PMC4794804; doi:10.1038/srep23104)
Supplement: Supplementary Information [file srep23104-s1.pdf]

Design of Advanced Photocatalysis System by Adatom Decoration in 2D Nanosheets of Group-IV and III-V Binary Compounds

Hao Jin[1], Ying Dai[2,*] and Bai-Biao Huang[2]

[1] *College of Physics and Energy, Shenzhen University, Shenzhen 518060, People's Republic of China*
[2] *School of Physics, State Key Laboratory of Crystal Materials, Jinan, 250100, People's Republic of China*

In **Table S1**, we have compared our results with others. Our results such as lattice constants, bond length, and bandgaps are in good agreement with previous work,[1-4] which indicate that the method employed in this work is reliable.

Table S1. The calculated lattice constant (a), nearest neighbor distance (d_x), and band gap (E_g) of XC (X=Si, Ge, Sn) and YN (Y=B, Ga) nano sheets

	SiC	GeC	SnC	BN	GaN
a (Å)	3.10<br>(3.07)[1]	3.26<br>(3.22)[1]	3.60<br>(3.55)[1]	2.51<br>(2.51)[1]	1.88<br>(1.85)[1]
d_x (Å)	1.79<br>(1.77)[1]	1.88<br>(1.86)[1]	2.08<br>(2.05)[1]	1.45<br>(1.45)[1]	3.26<br>(3.20)[1]
E_g (eV)	3.42<br>(3.88)[2]	3.21<br>(3.37)[2]	1.76<br>(2.0)[2]	5.69<br>(5.56)[3]	3.24<br>(3.40)[4]

[*] E-mail: daiy60@sina.com

As shown in **Fig. S1**, charge transfer across the heterointerface occurs on GeC-GeCH bilayer. A net charge gain at the GeC side has been identified with C-2*p* orbitals being the electron accommodator (see **Fig. 4** in manuscript).

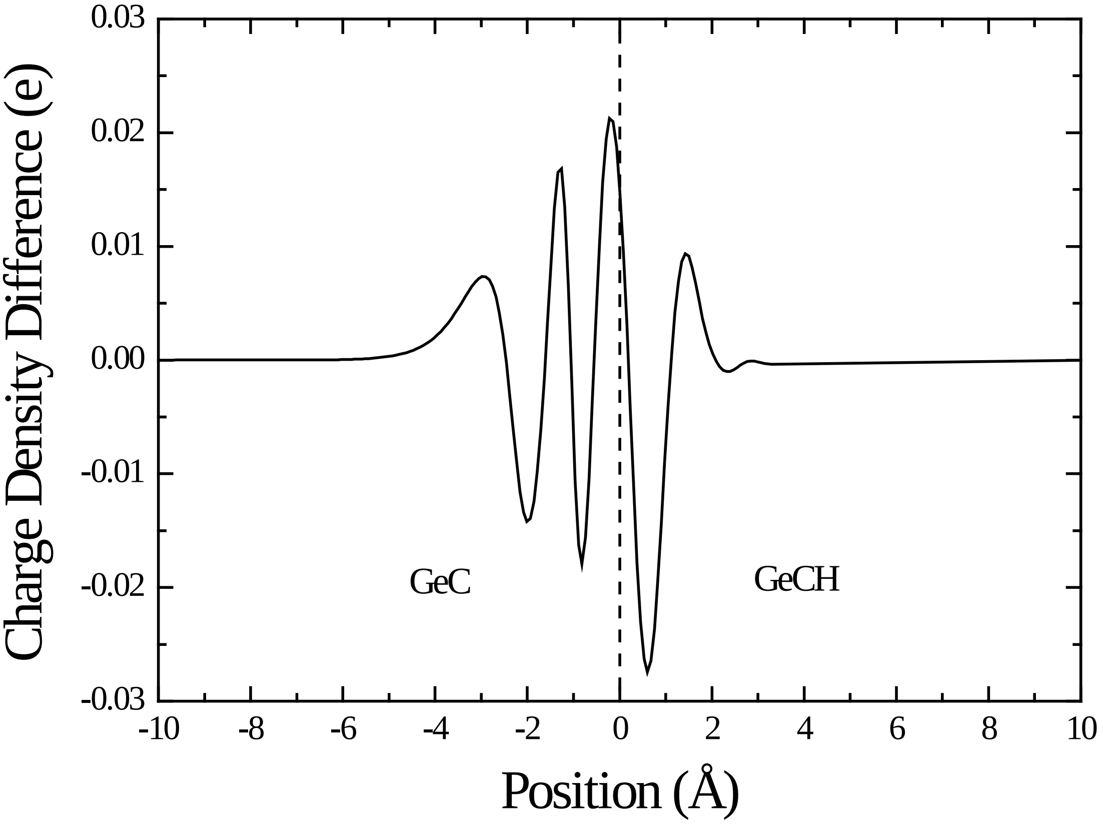

Fig. S1. Plane-averaged charge density difference of GeC-GeCH bilayer. The heterointerface is indicated by the dash line.

References:

1. Sahin, H. *et al.;* Phys. Rev. B 80, 155453 (2009).
2. Lv, T. *et al.;* J. Mater. Chem., 22, 10062–10068 (2012)
3. Song, L. *et al.*; Nano Letters 10, 3209–3215 (2010).
4. Xia, C. *et al.*; Acta Materialia, 61, 7720 (2013)
